# A Novel Type III Endosome Transmembrane Protein, TEMP

**DOI:** 10.3390/cells1041029

**Published:** 2012-11-05

**Authors:** Rajith N. Aturaliya, Markus C. Kerr, Rohan D. Teasdale

**Affiliations:** Institute for Molecular Bioscience, The University of Queensland, St. Lucia, Queensland, 4072, Australia; Email: raj.aturaliya@gmail.com (R.N.A.); kerr@mpiib-berlin.mpg.de (M.C.K.)

**Keywords:** endosome, subcellular localisation, membrane protein

## Abstract

As part of a high-throughput subcellular localisation project, the protein encoded by the RIKEN mouse cDNA 2610528J11 was expressed and identified to be associated with both endosomes and the plasma membrane. Based on this, we have assigned the name TEMP for Type III Endosome Membrane Protein. TEMP encodes a short protein of 111 amino acids with a single, alpha-helical transmembrane domain. Experimental analysis of its membrane topology demonstrated it is a Type III membrane protein with the amino-terminus in the lumenal, or extracellular region, and the carboxy-terminus in the cytoplasm. In addition to the plasma membrane TEMP was localized to Rab5 positive early endosomes, Rab5/Rab11 positive recycling endosomes but not Rab7 positive late endosomes. Video microscopy in living cells confirmed TEMP’s plasma membrane localization and identified the intracellular endosome compartments to be tubulovesicular. Overexpression of TEMP resulted in the early/recycling endosomes clustering at the cell periphery that was dependent on the presence of intact microtubules. The cellular function of TEMP cannot be inferred based on bioinformatics comparison, but its cellular distribution between early/recycling endosomes and the plasma membrane suggests a role in membrane transport.

## 1. Introduction

Determination of the subcellular distribution of individual proteins is essential to understanding a proteins function. Furthermore, for novel proteins for which a function cannot be inferred based on homology to other characterized proteins it is often one of the first traits investigated. Previously, we have performed several high-throughput subcellular localization projects on subsets of mouse proteins defined as part of the FANTOM project to determine the transcriptional output from the mouse genome [1,2,3]. For many of these proteins it represented the first property of these proteins to be determined and many displayed subcellular distributions of interest. All of the data captured from these projects was compiled and made publicly available on the LOCATE database [4,5,6]. Here we report the initial characterisation of a novel protein TEMP, a Type III Endosome Membrane Protein that displayed a plasma membrane and intracellular punctate subcellular localisation phenotype in the high throughput subcellular localisation screen [1]. Type III membrane proteins have a single membrane-spanning domain that acts as a reverse signal-anchor which results in the translocation of the amino-terminus across the membrane [7]. 

## 2. Results and Discussion

### 2.1. Bioinformatic Analysis of TEMP

**Figure 1:**
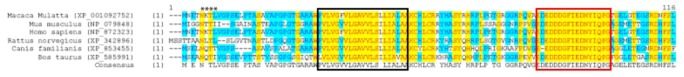
Multiple protein sequence alignment between *murine* TEMP and selected orthologs. A multiple sequence alignment was performed using the AlignX tool of VectorNTI using the default parameters. The following schema was used to disseminate different features: non-similar residues are shown in black type; conservative residues are highlighted blue and identical residues are highlighted yellow. The highly conserved transmembrane domain (black) and cytoplasmic motif (red) are boxed with the conserved N-glycosylation motif indicated with asterisks.

TEMP was originally discovered as part of RIKEN’s FANTOM3 project [8] as a predicted open-reading frame of 111 amino acids in the murine cDNA clone 2610528J11. Bioinformatic analysis identified that TEMP does not have any significant relationship to other proteins domains or families. However, TEMP is predicted to encode a single transmembrane domain [9] at residues 30–50 but no evidence of an N-terminal signal peptide was detected. BLAST analysis of the protein coding sequence of TEMP determined that orthologous protein sequences were only present in mammalian species. The multiple sequence alignment of these orthologous proteins (Figure 1) revealed that the transmembrane domain is highly conserved across all species with >80% sequence identity. The second highly conserved region near the carboxyl-terminus of the protein encodes a 15 amino acid sequence, EDDDGFIEDNYIQPG, that demonstrates 100% identity across all species. Interestingly, this sequence is also present in the Leucine-rich repeat containing Protein 19 (Lrrc19), a Type I transmembrane protein related to Toll-like receptors involved in the innate immune system [10]. This sequence is highly conserved within Lrrc19 othologs and is located within its cytoplasmic domain. The protein motif observed in both TEMP and Lrrc19 has not previously been characterised, however, the high degree of conservation of this sequence suggests functional importance. Other predicted motifs identified within TEMP and conserved across species included a putative glycosylation site within the amino-terminus and a putative endosome sorting [D/E]xxx[L/I] motif [11] at the carboxy-terminus [4,5,6]. 

The tissue-specific expression pattern of TEMP in mouse was examined using BioGPS/ SymAtlas [12,13]. TEMP demonstrates a restricted, tissue-specific expression profile to the stomach, kidney, large and small intestines and kidney at levels ten times higher than the median value of the transcript for all of the tissues examined. 

### 2.2. TEMP, is a Type III Transmembrane Protein

**Figure 2:**
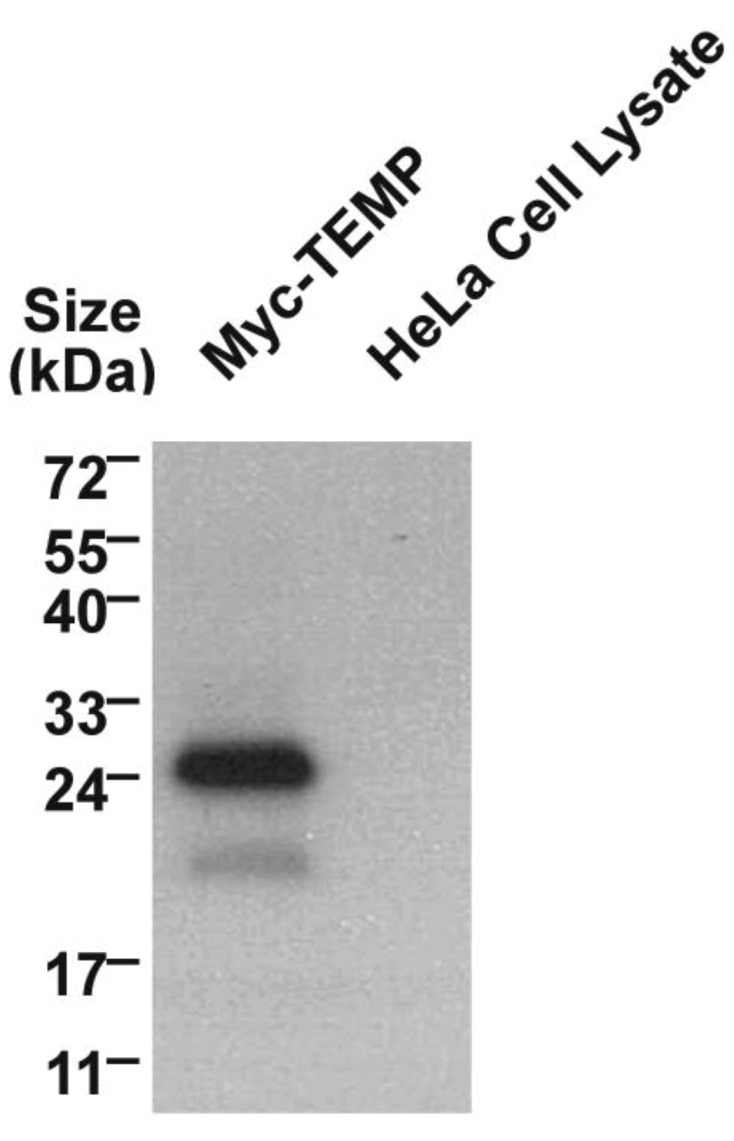
Western immunoblotting of myc-TEMP expression constructs.The full-length TEMP engineered to encode an amino-terminal myc-epitope was transiently transfected into HeLa cells and expressed for 24 h. Whole cell lysate were prepared and analysed using a 10% SDS-PAGE gel. Western immunoblotting to detect the myc epitope was performed. Untransfected HeLa whole cell lysates were run as a negative control.

**Figure 3:**
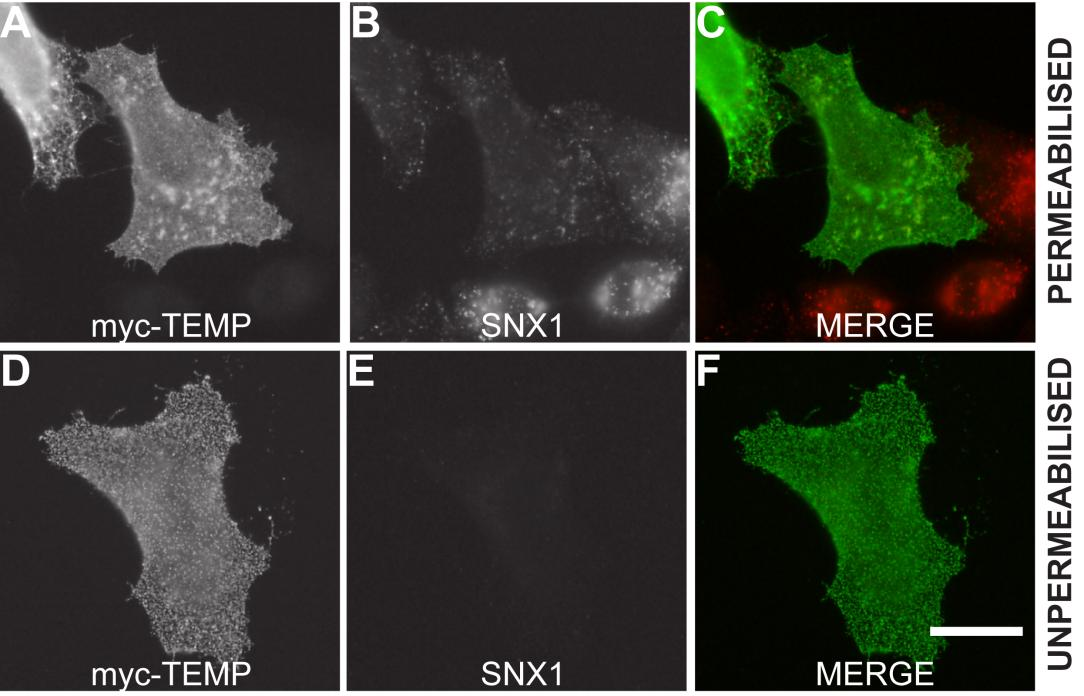
TEMP is a Type III membrane protein. Amino-terminal myc-tagged TEMP was transiently transfected into HeLa cells and expressed for 24 h. The exposure of TEMP’s amino-terminus to the extracellular environment was confirmed by analyzing non-permeabilised cells (Surface labeled) and permeabilised cells by indirect immunofluorescence using anti-myc antibodies. The inclusion of antibodies to the cytoplasmic SNX1 protein controlled for the integrity of the plasma membrane. Images were captured using a Zeiss LSM 510 META confocal laser scanning microscope. Bar represents 10 μm.

Mammalian expression plasmids with a myc epitope located at the amino-terminus of TEMP were transiently expressed in HeLa cells and the whole cell lysate was analysed using Western immunoblotting. TEMP has a predicted molecular mass of 11.5 kDa, increasing to 12.8 kDa with the myc-epitope, however the observed molecular mass of the expressed construct is 24 kDa (Figure 2). The observation of this larger molecular mass could be attributed to post-translational modification most likely N-glycosylation of the conserved site at the amino-terminus. To determine the topology of TEMP with respect to the membrane, the amino-terminal myc-tagged expression construct was transiently transfected into HeLa cells and the topology of the amino-terminus was then investigated in both permeabilised and unpermeabilised cells (Figure 3). An antibody against the sorting nexin 1 (SNX1) protein, a peripheral membrane protein that resides in the cytosol and associates with endosomal membranes [14], was used as an internal control to determine the integrity of the plasma membrane in individual cells. Detection of the myc epitope at the cell surface in unpermeabilised cells indicates the amino-terminus is exposed to the extracellular surface (Figure 3D). The plasma membrane of these surface labelled cells is uncompromised as is demonstrated by an absence of SNX1 labeling (Figure 3E) when compared to the cells permeabilised with 0.1% Triton X-100 where SNX1 labelling is clearly observed (Figure 3B). Collectively, these results indicate that the N-terminus of TEMP is expressed extracellularly. Combining this data with the computational prediction of a single transmembrane domain supports that TEMP has a Type III topology with respect to the plasma membrane [7]. This orientation is consistent with the amino terminus of the protein being exposed to the lumen of intracellular organelles and hence the conserved N-glycosylation motif would be available for post-translational modification. In addition, the motif shared with Lrrc19 would likewise be present in the cytoplasm along with the proposed carboxy-terminal endosome sorting motif. 

### 2.3. TEMP Extensively Colocalises with Early Endosomes and Recycling Endosomes

**Figure 4:**
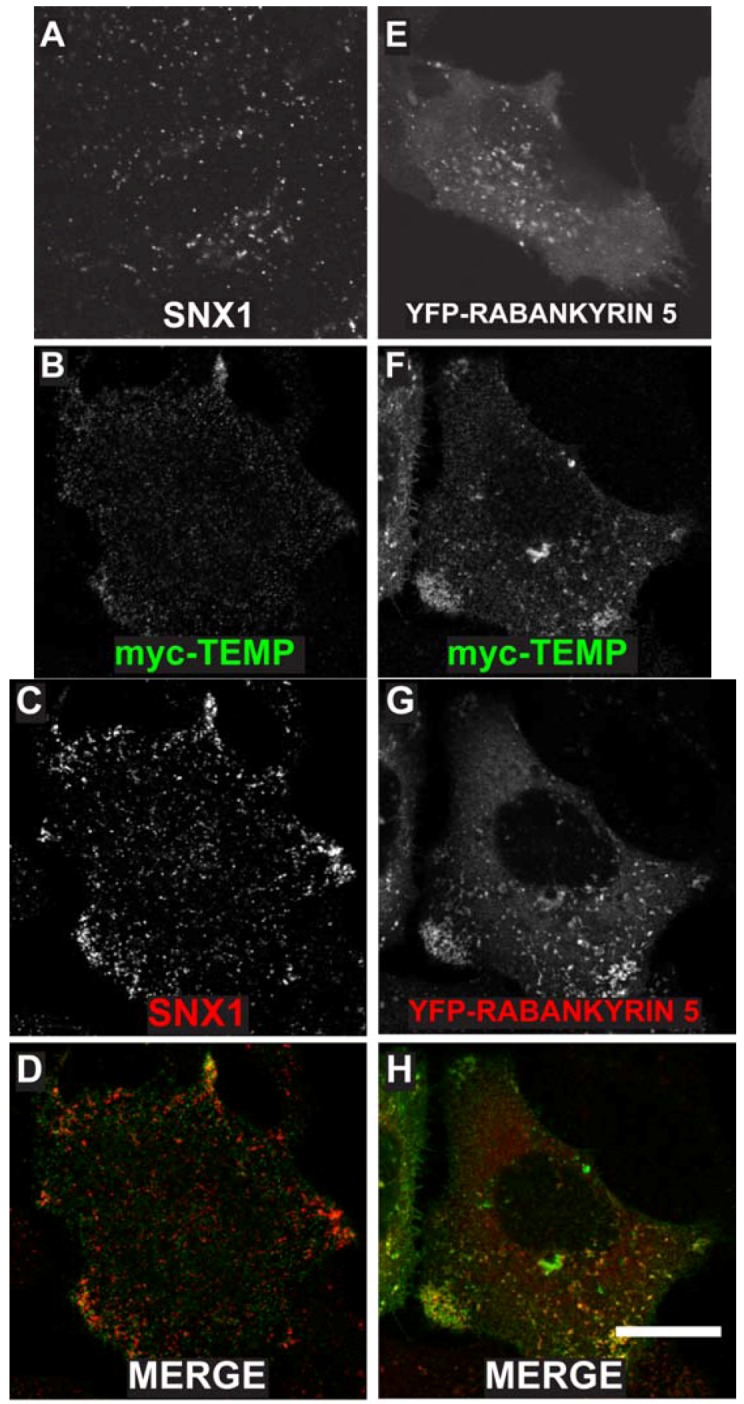
Expression of TEMP in HeLa cells results in the redistribution of a number of early endosomal markers. Sub-confluent HeLa cells were mock transfected (**A**), transiently transfected with myc-TEMP (**B**–**D**) or YFP-Rabankyrin-5 (**E**), or co-transfected with myc-TEMP and YFP-Rabankyrin-5 (**F**–**H**) and expressed for 24 h. Samples were subsequently fixed in 4% paraformaldehyde prior to permeabilisation and immunolabelling with anti-myc antibodies (Panels **B**, **F**) and SNX1 antibodies (Panels **A**, **C**). Images were subsequently captured using a Zeiss LSM 510 META confocal laser scanning microscope with appropriate band pass filter settings. Bar represents 10 μm.

TEMP was previously demonstrated to localise to the plasma membrane and to intracellular punctate structures using a linear, amino-terminal, myc-tagged expression construct [1]. To determine the nature of the intracellular compartments to which TEMP localises, further co-localisation studies were initially performed with two endosome proteins SNX1 and YFP tagged Rabankyrin-5. SNX1 is a peripheral membrane protein that regulates the correct sorting of receptors at the early endosome [14,15,16]. TEMP clearly co-localises with SNX1 positive structures (Figure 4B–D) demonstrating that it is present on early endosomes. Interestingly, cells expressing TEMP present a clear redistribution of SNX1 labelling with a larger concentration of puncta in the cell periphery (Figure 4C) when compared to untransfected cells (Figure 4A). Rabankyrin-5 is a Rab5 effector that is involved in the fusion of early endosomes [17]. TEMP also clearly co-localises with YFP-Rabankyrin-5, particularly in the cell periphery as is shown in Figure 4F–H. Again, similar to SNX1, the distribution of YFP-Rabankyrin-5 (Figure 4E) is altered upon co-expression with TEMP resulting in its redistribution from a diffuse punctate staining across the entire cell to be concentrated in the cell periphery (Figure 4G). This data demonstrates that TEMP clearly localises to a number of endocytic compartments with the primary evidence demonstrating TEMP is localised to early endosomes. Additionally, expression of TEMP appears to affect the distribution of the early endosomal markers presented. 

**Figure 5:**
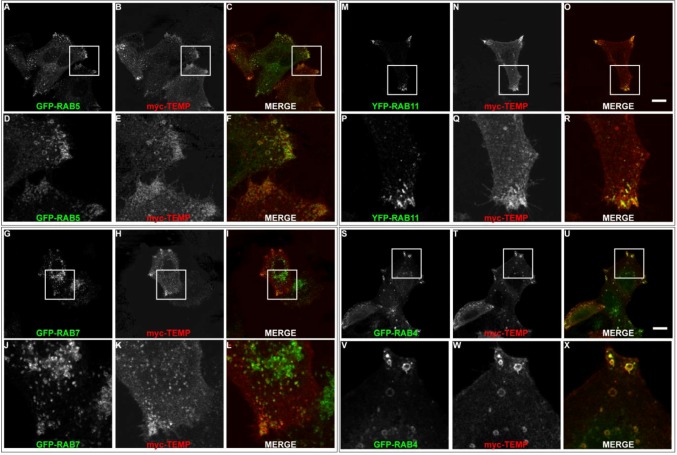
TEMP localises predominantly to early and recycling endosomes. Sub-confluent HeLa cells were transiently co-transfected with myc-TEMP (Panels B/E/H/K/N/Q/T/W) and GFP-Rab5 (Panels A/D), GFP-Rab7 (Panels G/J), GFP-Rab11 (Panels M/P) or GFP-Rab4 (Panels S/V), respectively, and expressed for 24 h. Samples were subsequently fixed in 4% paraformaldehyde prior to permeabilisation and labelling with an anti-myc antibody and appropriate secondary antibodies (as per Materials and Methods). Images were then captured using a Zeiss LSM 510 META confocal laser scanning microscope with appropriate band pass filter settings. Bar represents 10 μm.

Although TEMP demonstrates partial colocalisation to early endosomes, a large proportion of punctate structures fail to colocalise. To further investigate this observation colocalisation studies were performed with the Rab proteins to elucidate the precise endosomal sub-compartments to which TEMP localises. Rab proteins are GTPases that regulate membrane traffic and as such are localised to specific subcompartments (reviewed in [18,19,20]). Rab5 is required for transport from the plasma membrane to the early endosome [21,22] and represents a marker for early endosomes. TEMP was co-expressed with GFP-Rab5 (Figure 5A–F). TEMP demonstrates colocalisation with Rab5 in punctate vesicular structures at the periphery of the cell (Figure 5D–F). Overexpression of Rab5 alone can result in the dilation of endosome compartments due to the change in kinetics of subsets of membrane transport pathways [23]. Rab7 represents a well established marker for late endosomes [24]. TEMP was co-expressed with GFP-Rab7 (Figure 5G–L), however, only a few instances of co-localisation to the same structure were observed. Puncta that do demonstrate colocalisation are at the cell periphery (Figure 5J–L), however, the majority of Rab7 is observed in a perinuclear region and typically do not contain TEMP. The colocalisation of TEMP relative to Rab5 and Rab7 is consistent with it associating with early endosome with limited association with late endosomal compartments. To further refine the types of early endosomes TEMP associates with colocalisation studies with Rab4 and Rab11 were performed. Both of these Rabs have been implicated in recycling of the transferrin receptor to the plasma membrane and are markers of the recycling endosome [25,26,27,28,29]. Rab11 has been localised to perinuclear recycling endosomes and in trafficking from the plasma membrane to the Golgi apparatus [29]. TEMP clearly demonstrates colocalisation when co-expressed with YFP-Rab11 as is seen in Figure 5M-R. This colocalisation pattern is particularly apparent in the clusters of endosomes within the cell periphery (Figure 5P–R). Rab4 has been localised to early and recycling endosomes and has been implicated in the sorting of proteins at this stage of endocytosis [25,26,28]. TEMP clearly demonstrates colocalisation when co-expressed with GFP-Rab4 as is observed in Figure 5S–X. Colocalisation is strikingly apparent in enlarged endosomes at the cell periphery (Figure 5V–X). Collectively this subcellular localisation indicates that TEMP associates with both early and recycling endosomes. 

### 2.4. TEMP Associates with Endosomal Tubular-Vesicular Structures in Living Cells

**Figure 6:**
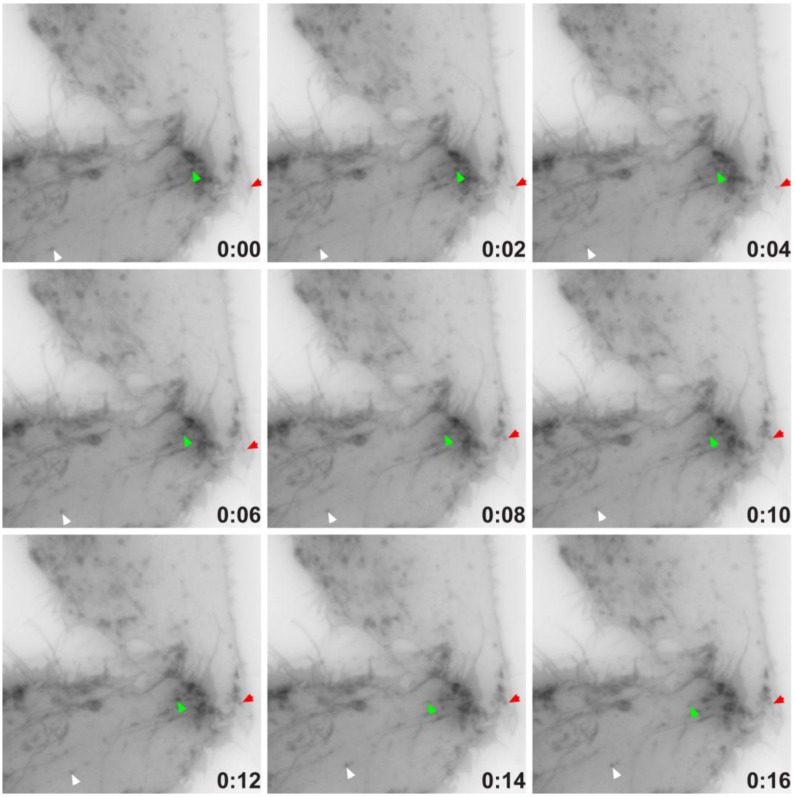
Time-lapse video microscopy of TEMP-GFP. Sub-confluent HeLa cells grown on 35 mm glass bottom tissue culture dishes (MatTek) were transiently transfected with TEMP-GFP and expressed for 24 h. Cells were then washed with PBS and then placed into supplemented CO_2_-independent media. Time-lapse video microscopy was then performed using the 60X objective on an Olympus IX-81 OBS epifluorescence microscope attached to a heated chamber at 37 °C. This montage represents a 16 seconds window of a movie captured at one frame every 2 seconds for a total duration of 3 min. See Supplementary Movie 1.

**Figure 7:**
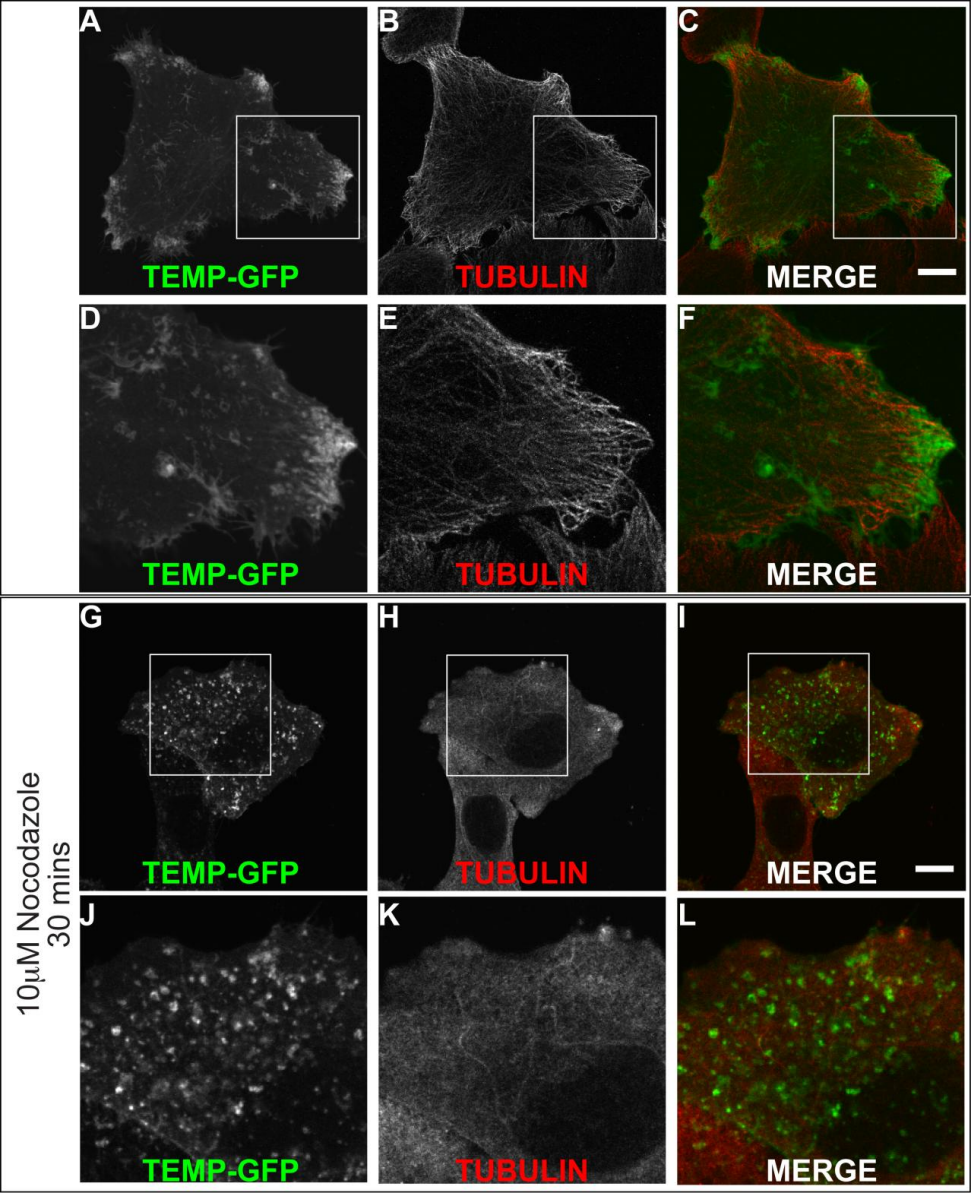
The peripheral concentration of endosomes containing TEMP is dependent upon the microtubule network. Subconfluent HeLa cells were transiently transfected with TEMP-GFP and expressed for 24 h. Samples presented in Panels G-L were treated with 10 μM nocodazole for 30 min at 37 °C to disrupt the microtubule cytoskeleton. Cells were subsequently fixed in 4% paraformaldehyde prior to immunofluorescent detection with anti α tubulin antibodies (Panels B/E/H/K) and appropriate secondary antibody labelling. Images were then captured using a Zeiss LSM 510 META confocal laser scanning microscope with appropriate band pass filter settings. Bar represents 10 μm.

We identified that TEMP clearly localises to early and recycling endosomes in fixed cells. To investigate it within live cells we generated a carboxyl-terminal GFP-tagged expression construct. Time-lapse videomicroscopy was performed to determine the dynamic nature of TEMP’s subcellular localisation in transiently transfected HeLa cells. As in fixed cells, TEMP is present on early-/recycling-endosomes concentrated at the cells periphery. In addition, in living cells TEMP was observed on numerous smaller vesicular structures that are observed moving throughout the cell (Figure 6). TEMP-GFP was also clearly localised to tubular endosome structures. It is evident from the time-lapse videomicroscopy that some of these tubular structures are dynamic whereas others appear to be more static in their nature. The number and nature of the tubules appears to vary from cell to cell. More dynamic tubules are observed trafficking from the perinuclear region to the cell periphery and vice versa. To determine if the microtubule network is responsible for the peripheral nature of these clusters of endosomes, HeLa cells were transiently transfected with TEMP and expressed for 16 h prior to 10 μM nocodazole treatment, subsequent fixation and immunodetection of the microtubule network (Figure 7). The disruption of the microtubule network altered the subcellular localisation of TEMP with loss of the concentration of peripheral endosomes, resulting in their even redistribution throughout the cytoplasm (Figure 7G/J). 

## 3. Experimental

### 3.1. Bioinformatics

General sequence characteristics including length, predicted InterPro domains and predicted transmembrane domains were collated from the LOCATE database [5,6]. Independent predictions were obtained using TMHMMv2.0 [30] for transmembrane domains, SignalP3.0 [31] for signal peptides and InterProScan [32,33,34] for the prediction of protein domains. Further details including putative glycosylation sites were obtained from the University of California Santa Cruz (UCSC) proteome browser [35,36]. SymAtlas developed by the Genomics Institute of the Novartis Research Foundation (GNF) [12] was used to analyse the gene expression patterns of the proteins investigated. Orthologs were analysed by submitting the query sequence to the BLAST tool [37,38]. The sequences were analysed against the non-redundant dataset provided by NCBI and corresponding orthologs were identified. These orthologs were subsequently aligned in a multiple sequence alignment using the AlignX tool in VectorNTI^®^ (Invitrogen, USA) using the default parameters.

### 3.2. Generation of Amino-Terminal Myc-Tagged Expression Constructs

Expression constructs comprised of an amino-terminal cytomegalovirus (CMV) promoter, an amino-terminal, myc-tagged protein coding sequence (CDS) and two C-terminal Simian Virus 40 (SV40) poly-adenylation sequences were generated using the Megaprimer PCR protocol outlined in [1]. The subsequent PCR product was subsequently, T-A cloned into pGEM^®^-T (ProMega, USA) according to the manufacturer’s instructions.

### 3.3. Generation of C-terminal GFP-tagged Expression Constructs

RIKEN clone 2610528J11 (PA76377.1) was used as a Template to amplify the open reading frame using primers 5'-CCC ATC CCG AAT TCG CCA CCA TGA TTG GAG GAA ACA C-3' and 5'-CCC ATC CCG GAT CCG GGA GAG AGA AGT GGT CCC G-3' which incorporate a 5' EcoRI site and a 3' BamHI site to the PCR product. The purified PCR product and pEGFP-N1 (Clontech Laboratories Inc., USA) were digested with EcoRI (New England Biolabs, USA) and BamHI (New England Biolabs, USA) and the subsequent products were ligated together using T4 DNA ligase (Invitrogen, USA).

### 3.4. Plasmids

GFP-Rab5 [25], YFP-Rab11 [25], GFP-Rab7 [39], YFP-Rabankyrin-5 [17] and GFP-Rab4 (kindly donated by Dr Frederic Meunier, The University of Queensland). 

### 3.5. Antibodies

Mouse monoclonal antibodies were used to detect the myc-epitope tag (Cell Signalling Technology, USA). Alternatively, a rabbit polyclonal antibody was used to detect the myc-epitope tag (Cell Signalling Technology, USA). Monoclonal mouse antibodies used included anti-α-tubulin (clone DM1A, mouse ascites fluid) (Sigma-Aldrich, USA), anti-Sorting Nexin 1 (SNX1) (BD Transduction Laboratories, USA). The following secondary antibodies were used for immunofluorescent labelling: goat anti-rabbit Alexa Fluor 488 (Molecular Probes, USA), goat anti-mouse Cy3 (Molecular Probes), goat anti-mouse Alexa Fluor 488 (Molecular Probes, USA) and goat anti-mouse Alexa Fluor 647 (Molecular Probes, USA).

### 3.6. Cell Culture and Transfection

HeLa cells and MDCK cells were cultured in Minimum Essential Medium (Eagle) (Life Technologies Inc., USA) supplemented with 2 mM L-glutamine and Earle’s BSS adjusted to contain final concentrations of 1.5 g/L sodium bicarbonate, 0.1 mM non-essential amino acids, 1.0 mM sodium pyruvate and 10% fetal bovine serum in 5% CO_2_ and 95% air at 37 °C. Subconfluent cell monolayers were transiently transfected with expression constructs using Lipofectamine 2000 (Life Technologies Inc., USA) and OptiMem (Life Technologies Inc., USA) as per the manufacturer’s instructions. 

### 3.7. Western Immunoblotting

Proteins were run on 10% Sodium Dodecyl Sulfate Poly-Acrylamide Gels (SDS-PAGE) gels prior to transfer onto PVDF membranes (Millipore, USA) according to the manufacturer’s instructions. Immunoblotting was subsequently performed as described in [40]. Anti-myc antibodies were used to detect the epitopes in conjunction with sheep anti-mouse Ig horseradish peroxidase conjugated secondary antibodies (Silenus, Australia).

### 3.8. Surface Labelling Immunofluorescence Protocol (Unpermeabilised cells)

Coverslips of sub-confluent HeLa cells were rinsed with ice-cold CO_2_ independent media (Life Technologies Inc.) and then placed on a drop of ice-cold CO_2_ independent media supplemented with 0.1% BSA on ice for 5 min. Cells were then placed on 30 μL of primary antibody diluted in ice-cold CO_2_ independent media/0.1% BSA for 30 min at 4 °C prior to fixation with 4% paraformaldehyde for 20 min at room Temperature. Cells were then washed three times with PBS prior to quenching with 50mM ammonium chloride in PBS for 15 min at room Temperature. Cells were then incubated in blocking solution composed of 0.2% BSA and 0.2% fish skin gelatin in PBS for 10 min at room Temperature prior to further incubation with 25 μL of the secondary antibody in blocking solution for 30 min at room Temperature. Cells were then washed thrice in PBS prior to mounting with MO-WIOL. 

### 3.9. Indirect Immunofluorescence

Indirect immunofluorescence was performed as described [1]. Cell monolayers were treated with 10 μM nocodazole (Sigma-Aldrich, USA) for 30 min prior to fixation. 

### 3.10. Confocal Microscopy

Representative images of the observed localisation patterns for all fixed cell experiments were performed on a Zeiss Axiovert 200 M SP LSM 510 META (Zeiss, Germany) inverted, laser scanning confocal microscope with appropriate band pass filter settings. Data was analysed using the LSM 510 META (Zeiss, Germany) software and images were prepared using Adobe Photoshop 7.0 (Adobe Systems, USA).

### 3.11. Time-Lapse Videomicroscopy

Cells were prepared in MatTek glass bottom tissue culture dishes (MatTek Corporation, USA) and grown under conditions as previously described. Prior to analysis with time-lapse videomicroscopy, cells were washed with PBS, and placed into CO2-independent media (Invitrogen, USA) supplemented with 5% fetal calf serum and 2 mM L-glutamine (Life Technologies Inc., USA). Videomicroscopy was then performed using an Olympus IX-81 OBS epifluorescence microscope attached to a heated chamber (Solent Scientific, UK) at 37 °C. Images were captured with the 60X objective at varying exposure times and intervals using Cell^R software (Olympus, Japan). ImageJ software [41] was used to process image stacks. 

## 4. Conclusions

TEMP is a single domain transmembrane protein with a type III membrane topology, namely with the amino-terminus on the extracellular or lumenal face, and the carboxy-terminus in the cytoplasm. Bioinformatic characterisation failed to infer any functional insights into the protein as TEMP lacks any known functional domains nor does it have homology to any proteins of known function, thus representing a completely novel, uncharacterised protein. Initially we nominated to characterise its subcellular localization. Analysis of fixed cells and dynamic time-lapse videomicroscopy revealed that TEMP localises predominantly to the plasma membrane and to early and recycling endosomes. Truncation of the carboxy-terminus of TEMP to remove the putative [DE]XXX[LI] endosome sorting motif did not alter the distribution of the TEMP between endosomes and the plasma membrane (data not shown). Therefore the sorting signal or cellular mechanism responsible for TEMP’s endosome localization needs to be identified. The transient expression of TEMP in HeLa cells resulted in the redistribution of Rab4 and Rab11 positive recycling endosomes and Rab5 positive early endosomes to the periphery of the cell. Recycling endosomes have been reported to have a highly variable morphology with a tubulovesicular phenotype which is proposed to facilitate rapid and dynamic trafficking events [42,43,44], consistent with the tubular structures observed. The observed clustering of endosomes to the peripheral regions of the cell when TEMP is overexpressed represents an unusual and interesting phenotype in membrane trafficking. This phenotype could be explained by modulation of a number of different membrane transport pathways including from the early endosome to the late endosome. TEMP containing peripheral endosomes clusters are redistributed when the microtubule network is disrupted. It has been demonstrated that the microtubule network is necessary for trafficking from early endosomes to Rab11 positive recycling endosomes [45]. Thus, it is possible that Nocodazole treatment may have disrupted the trafficking of TEMP from early endosomes to recycling endosomes resulting in more diffuse punctate structures. Alternatively, TEMP could have a role in regulating the rate of trafficking events that coordinate the recycling of endosomes to the plasma membrane. A number of key factors are involved in the recycling of endosomes back to the cell surface, one of which is ADP-ribosylation factor 6 (Arf6) [46,47,48] which has been shown to play a number of important roles in the cell including endocytosis, regulation of actin remodelling, regulation of membrane remodelling and endosome recycling to the cell surface [49,50,51,52]. Activation of Arf6-GTP occurs via Guanine- nucleotide Exchange Factors (GEFs) and hydrolysis of the GTP to GDP occurs via GTPase-Activating Proteins (GAPs). The cycling of Arf6 between active and inactive forms is essential for the process of recycling endosomes back to the plasma membrane. Over-expression of TEMP may, directly or indirectly, disrupt the endogenous equilibrium of the GEFs and GAPs, thereby preventing the recycling endosome from continuously cycling back to the plasma membrane. 

In conclusion, this study has expanded our understanding of a novel type III membrane protein, named TEMP, which localised to the plasma membrane, Rab5 positive early endosomes and Rab4/Rab11 positive recycling endosomes. TEMP’s subcellular localisation suggests a role for the protein in membrane trafficking between endosomes and the plasma membrane. Furthermore its association with the recycling endosome combined with its tissue specific expression pattern in polarised cell may indicate a function in maintaining cell polarity via the trafficking through the recycling endosome.

## Supplementary Files

Supplementary File 1 MOV-Document (MOV, 161 KB)
